# Student characteristics, professional preferences, and admission to medical school

**DOI:** 10.3205/zma001082

**Published:** 2017-02-15

**Authors:** Iris Kesternich, Heiner Schumacher, Joachim Winter, Martin R. Fischer, Matthias Holzer

**Affiliations:** 1 University of Leuven, Department of Economics, Leuven, Belgium; 2 University of Munich, Volkswirtschaftliche Fakultät, Seminar für Empirische Wirtschaftsforschung, Munich, Germany; 3 Klinikum der Universität München, Institut für Didaktik und Ausbildungsforschung in der Medizin, München, Germany

**Keywords:** admission to medical school, specialization choice, physician shortages, rural work

## Abstract

**Objectives:** A potential new avenue to address the shortage of country doctors is to change the rules for admission to medical school. We therefore study the link between high-school grade point average and prospective physicians’ choice to work in rural areas. To further inform the discussion about rules for admission, we also study the effects of other predictors: a measure of students’ attitudes towards risk; whether they waited for their place of study (*Wartesemester*); whether their parents worked as medical doctors; and whether they have some practical experience in the medical sector.

**Methods: **We conducted two internet surveys in 2012 and 2014. In the first survey, the sample comprised 701 students and in the second, 474 students. In both surveys, we asked students for their regional preferences; in the 2014 survey, we additionally asked students for their first, second, and third preferences among a comprehensive set of specializations, including becoming a general practitioner. In both surveys, we asked students for basic demographic information (age and gender), their parents’ occupation, a measure of subjective income expectations, a measure of risk attitudes, and their high-school grade point average (*Abiturnote*), and First National Boards Examination grade (*Physikum*). In 2014, we additionally asked for waiting periods (*Wartesemester*) as well as for prior professional experience in the health-care sector.

**Results: **We find that three factors increase the probability of having a preference for working in a rural area significantly, holding constant all other influences:

1. having a medical doctor among the parents,
2. having worse grades in the high-school grade point average, and
3. being more risk averse.

Moreover, we find that those willing to work in the countryside have significantly more experience in the medical sector before admission to medical school.

**Discussion: **Our results suggest that a change in the selection process for medical school may increase the supply of country doctors. Instead of focusing on the high-school grade point average, universities could even more intensely screen for study motivation through interviews or by taking into account students’ background, extracurricular activities, or waiting periods.

## Zusammenfassung

**Forschungsziele: **Die Veränderung der Zugangsregelungen zum Medizinstudium stellt eine Möglichkeit dar, um die Knappheit von Landärzten zu verringern. Wir analysieren daher den Zusammenhang zwischen dem Abiturnotendurchschnitt und der Entscheidung zukünftiger Ärzte, in ländlichen Regionen zu arbeiten. Zur Anregung der Debatte über die Zulassungsregelungen beschäftigen wir uns zudem mit den Effekten anderer Einflussvariablen wie der Risikobereitschaft der Studierenden, der Anzahl der Wartesemester, der Zugehörigkeit der Eltern zum ärztlichen Berufsfeld sowie der praktischen Erfahrung der Studienbewerber im Gesundheitssektor.

**Methoden:** In den Jahren 2012 und 2014 führten wir zwei Internetbefragungen durch. Die erste Befragung umfasste 701 Studierende, die zweite 474 Studierende. In beiden Erhebungen wurden die Studierenden nach ihren regionalen Präferenzen für ihre künftige Berufsausübung gefragt; in der Befragung des Jahres 2014 ermittelten wir zudem die ersten, zweiten und dritten Präferenzen bezüglich der Facharztwahl. In beiden Befragungen wurden demographische Merkmale (Alter und Geschlecht), die Berufe der Eltern, subjektive Einkommenserwartungen, die Risikobereitschaft sowie die Notendurchschnitte in Abitur und Physikum erhoben. 2014 fragten wir darüber hinaus nach der Anzahl der Wartesemester und vorherigen praktischen Erfahrungen im Gesundheitssektor.

**Ergebnisse: **Drei Faktoren erhöhen ceteris paribus die Wahrscheinlichkeit einer Präferenz für eine Beschäftigung in ländlichen Gebieten signifikant:

1. ein Elternteil ist Arzt,
2. relativ schlechtere Abiturnoten und
3. geringere Risikobereitschaft.

Des Weiteren zeigt sich, dass Studierende, die eine Tätigkeit auf dem Land anstreben, vor der Zulassung zum Medizinstudium signifikant häufiger eigene praktische Arbeitserfahrungen im Gesundheitssektor haben.

**Diskussion: **Unsere Ergebnisse legen nahe, dass eine Veränderung des Auswahlprozesses für das Medizinstudium das Angebot an Landärzten erhöhen könnte. Statt sich nur auf die Abiturnote zu konzentrieren, sollten die Universitäten die Motivation für das Studium in größerem Maße berücksichtigen, sei es mit Hilfe von Auswahlgesprächen oder durch die Berücksichtigung von Herkunft, außerschulischen Aktivitäten oder Wartesemestern.

## 1. Introduction

Countries throughout the world – both developed and developing – struggle with imbalances in their physician work force, in particular with shortages of general practitioners and of doctors in rural areas. These imbalances have resulted in a large body of research that analyses medical students’ specialization and location choices and policy measures designed to alleviate physician shortages, see [1]. Research on these issues is also encouraged by the World Health Organization (WHO) through a program of work aimed at increasing supply of health workers in remote and rural areas [http://www.who.int/hrh/migration/retention/en/ accessed 9 August 2014]. Among the factors that have been shown to affect whether students are willing to work in rural areas are monetary [2], and non-monetary job attributes such as control over working hours or the possibility to work part-time [3], and medical students’ preferences [4].

In Germany, there are strong regional imbalances in the physician workforce, despite the fact that practice licenses are awarded on the basis of regional quota. Physician density in rural areas, especially in Eastern Germany, is low. In particular, there is a shortage of general practitioners [5]. This is a major concern for health policy as general practitioners play a central role in health care provision in rural areas. Consequently, there is an ongoing discussion in Germany about whether candidates who want to become rural practitioners should be provided with easier access to medical studies. According to an estimate of the National Association of Statutory Health Insurance Physicians (KVB), more than 66,000 general practitioners will retire until the year 2020, which must be compared to a total of about 9,000 physicians newly coming from university each year [6].

The goal of this paper is to inform this discussion with some recent evidence from two online surveys among medical students of two large German medical faculties. A potential new avenue to address the shortage of country doctors is to change the rules for admission to medical school. We therefore study the link between high-school grade point average and prospective physicians’ choice to work in rural areas. To further inform the discussion about rules for admission, we also elicit whether students waited for their place of study (*Wartesemester*), whether students’ parents worked as medical doctors, and whether students have some practical professional experience in the medical sector.

Our discussion focuses on the potential effects of entry requirements into medical schools (such as a threshold based on the high-school grade point average). If the high-school grade point averages of applicants who consider and those who do not consider rural careers have a different distribution, changing the entry threshold should change the shares of these two types among the admitted students. Another contribution of our study is that the statistical analysis allows for potential correlations between the choices of becoming a primary care physician and of working in the country-side.

## 2. Methods

### Survey administration and samples

In 2012 and 2014, we conducted two online surveys among large samples of students from two large German universities, the University of Munich (LMU) and the Technical University of Munich (TUM). The surveys were administered by CentERdata Tilburg, Netherlands, and the medical faculties of both universities. We received the data with any personal identifiers removed; this was communicated to the participants before they took part in the survey. 

A request for approval of the surveys was placed with the ethics commission of the Medical Faculty of the University of Munich. The ethics commission issued a declaration of no-objection (UE Nos. 260-12 and 540-14).

In 2012, about 2.800 medical students were enrolled for their clinical studies (years 3 to 6) at the two universities when our survey fieldwork took place; in 2014, this number was 3098 due to an increased intake. For both surveys, we sent out e-mails to all these students inviting them to participate in our study. In 2012 and 2014, 701 and 474 students participated, respectively. 

#### Dependent and independent variables

##### Dependent variables

In both surveys, we asked students for their location preferences – working in an urban area, in a rural area, or abroad. We consider these three choices separately in the descriptive analysis; in the multivariate analysis we combine “urban” and “abroad” into one category that is then compared with “rural”. In the 2014 survey, we additionally asked students for their first, second, and third preferences among a comprehensive set of specializations, including becoming a general practitioner.

##### Independent variables

In both surveys, we asked students for basic demographic information (age and gender), their parents’ occupation, a measure of subjective income expectations, a measure of risk attitudes, and their high-school grade point average (*Abiturnote*), and their First National Boards Examination grade (*Physikum*). In 2014, we additionally asked for waiting periods (*Wartesemester*) as well as prior experience in the health-care sector (e.g., internships).

#### Details on measures and transformations

In Germany, school grades are measured on a scale from 1 (excellent) to 6 (fail). For our regression analysis, we standardize the high-school grade point measure by subtracting its mean and dividing by its standard deviation to obtain a variable with mean zero and standard deviation one.

We elicited the income expectations of medical students by asking five questions of the form “What do you think is the percent chance that your net income (after deductions and taxes) five years after finishing your studies will be less than X Euros per month?” Through the sequence of these five questions, the amount X increased. This method allows us to estimate the mean of students’ subjective distribution of future income using a statistical algorithm that approximates this distribution non-parametrically [7], [8].

We are also interested in risk aversion because it has been shown to be an important determinant of the decisions to become self-employed and to take up a profession with (relatively) high income variability. We ask students whether they are willing to take risks on a scale from zero to ten where zero means “not at all” and ten means “very willing to take risks.” This measure was created for the German Socioeconomic Panel, and it has been validated using both experimental measures of risk preferences as well as actual decisions in the field [9]. We use this measure to create a binary variable that indicates whether students are in the upper or lower half of their peers regarding risk aversion.

#### Statistical methods

We begin by testing for differences in the means of the independent variables across the values of their location preference using* t*-tests. 

Next, we conduct multivariate regression analyses in order to assess the effect of the independent variables in a* ceteris paribus* sense, which also accounts for multiple testing. As the dependent variables are dichotomous, we estimate probit models. Because of the great importance of general practitioners for serving rural areas, we analyze the decision to work on the countryside and the decision to become a general practitioner jointly by estimating a bivariate probit model. 

As the second dependent variable was elicited only in the 2014 survey, we restrict all multivariate regressions to this sample. 

Male, parents physicians, high-school grade point average (*Abiturnote*), the First National Board Examination grade (*Physikum*), mean income expectations, risk aversion, waiting time, and experience in the medical sector denote the covariates which affect both dependent variables, the choices of becoming a rural and a general practitioner, respectively. We estimate both equations simultaneously using the bivariate probit method, which assumes that the error terms are jointly normally distributed with zero means, variances of one, and a correlation coefficient that can be estimated as a parameter along with the coefficients of the covariates [10]^1^. 

We use a bivariate probit model for the following reasons: First, univariate probit or logit models permit statements about the influence of the independent variables on both dependent variables separately. For example, they would permit conclusions on how being male influences either the probability of becoming a general practitioner or the probability of practicing in a rural area. A bivariate probit model also allows us to assess such hypotheses – in addition, it allows us to test hypotheses on how the independent variables influence the *joint probability* of working as general practitioner and practicing in a rural area at the same time. 

Second, in the bivariate probit model it is straightforward to test whether there is a correlation in the unobserved factors in the two equations (i.e., for the decisions to work in a rural area and the decision to specialize as a general practitioner). The bivariate probit model also reveals whether this correlation is positive or negative. 

## 3. Results

In the 2014 (2012) survey, about 15.9 (17.7) percent of students stated that they intend to work in a rural area in Germany, 69.1 (66.9) percent intended to work in a city in Germany, and 15.03 (15.05) percent intended to work abroad. In the 2014 survey, 10.2 percent of students responded that their first job preference is to become a general practitioner. In the data from the 2014 survey, the correlation between the indicators for those who want to work in rural areas and those who want to become a general practitioner is 0.202.

Table 1 (Tab. 1) contains descriptive statistics and verbal definitions of all variables. We are mainly interested in differences in the means of the independent variables between those students who want to practice in a rural area in Germany and all other students (comparison group). Compared to all others, those willing to practice in rural areas have worse, but still very good, grades (two-sided *t*-tests, *p*=0.063 in 2012 and *p*=0.026 in 2014).

Students willing to practice in a rural area also waited longer for a place to study medicine, but this difference is not significant at the 10 percent level using a two-sided *t*-test. They also gathered considerably more practical experience in the medical sector before taking up their studies (this question was only asked in 2014; two-sided* t*-test, *p*=0.072). Their parents are significantly more often physicians themselves. The difference is significant at the 10 percent level only for 2014 (two-sided *t*-test, *p*=0.051). Students who want to practice in rural areas are also significantly more risk averse than their fellow students (two-sided *t*-tests, *p*=0.054 in 2012 and *p*=0.012 in 2014).

Income expectations of students willing and not willing to practice in the countryside are surprisingly similar: In 2014, there are no significant differences between both groups. Students who want to work in rural areas and those who do not want to work in rural areas both expect to earn about 4400 Euro per month after taxes and deductions, and even the estimated variances of students’ income expectations are similar in these two groups. In 2012, income expectations were somewhat lower for those students intending to practice in a rural area, but differences were small (about 4200 Euros for those who want to practice in rural areas and 4300 Euros for all others).

Regarding the third group of students, those who consider going abroad, there are few significant differences to other students, with one notable exception: They tend to have better grades in their high school point diplomas. The difference is statistically significant in 2012 (two-sided *t*-test, *p*=0.005); in 2014, the difference is smaller and not significant at the ten percent level when using a two-sided *t*-test. Consequently, those students who consider going abroad have also experienced waiting periods significantly less often before being admitted as medical students (this question was only asked in 2014; two-sided *t*-test, *p*=0.058). 

Next, we analyze all measured determinants of the choice to become a rural practitioner jointly using multivariate regression (which also takes account of multiple testing). As explained above, we have two dichotomous dependent variables, and thus estimate a bivariate probit model. 

The bivariate probit model allows us to estimate the effect on the two outcomes (working a rural area and working as general practitioner) separately, but also to estimate what influences the joint probability to practice as general practitioner in a rural area. In Table 2 (Tab. 2) we first consider the choices separately. We report marginal effects at the mean of the dependent variables (and for dichotomous variables, we report the effect for a change from zero to one). 

We find that three factors increase the probability of having a preference for working in a rural area significantly, holding constant all other influences:

1. having a medical doctor among the parents,
2. having worse grades in the high school grade point average, and
3. being more risk averse.

Interestingly, we find that two of the variables that increase the probability of having a preference for working in a rural area also predict a preference for becoming a general practitioner – namely, having a parent who works as a doctor and being risk averse.

Regarding the size of the marginal effects, it is interesting to see them in relation to the probability of having preferences for becoming a general practitioner (10.2 percent of all students) and for practicing in a rural area (15.9 percent). Having a physician among the parents makes it 56.9 percent more likely to state “general practitioner” as the preferred specialization and 47.8 percent more likely to plan to practice in a rural area. Having grades that are one standard deviation worse than the average makes it 28.3 percent more likely to plan to practice in a rural area. And last, being risk averse makes the general practitioner choice 55 percent and the rural choice 64.8 percent more likely. 

In Table 3 (Tab. 3), we consider the joint probability of having a preference for working as general practitioner and in a rural area. We find that four factors increase this probability, holding constant all other influences: being female (plus 21.7 percent), having a medical doctor among the parents (plus 30.4 percent), having worse grades in the high school grade point average (plus 12 percent), and being more risk averse (plus 34.8 percent).

We note that the error terms of the two regression equations that are jointly estimated in this bivariate probit model are significantly positively correlated (see the estimate in the last line of Table 3 (Tab. 3)). Thus, there are also unobserved factors that determine both preferences jointly and in the same direction. The substantive implication is that students have a common taste for these two professions. In a statistical sense, this result confirms that the two outcome equations should indeed be estimated jointly rather than using two separate probit or logit models. 

## 4. Discussion

The results on entry grades and self-assessed ability as well as job attributes provide several insights into why students choose to become a country doctor. 

Perhaps the most interesting result is that high-school grade point averages are lower among students intending to work in rural areas. This suggests that lowering admission requirements for medical school might increase the share of students willing to work in rural areas. However, this would inevitably increase the costs of education and the overall supply of physicians. Such a measure is unlikely to be accepted by policy makers and the medical profession. In the following, we discuss two instruments that may alleviate the imbalance in location choice and keep the total number of students (and physicians) constant.

The first policy instrument is multi-dimensional admission requirements. Medical schools could select applicants by considering not only their high-school grade point average but also their study motivation. In particular, it should be made easier for those willing to pursue a rural career to enter medical school. Motivational screening would have to be an important part of the admission process.

A promising source of information about study motivation could be an applicant’s curriculum vitae, see [11]. A number of activities indicate an intrinsic motivation to become a health worker (e.g., [12]), for example the completion of a voluntary social year; activities in social projects with children, elderly people or disabled persons; and voluntary work in religious communities. Medical schools could facilitate access for those applicants who are engaged in these activities, but have relatively low grades. In many institutions and disciplines, this type of motivational screening is already used to select students.

An alternative source of information about study motivation could be an interview with the applicant. Multiple mini-interviews (MMIs) are already used for the selection of medical students. It typically consists of several stations with different interviewers, each lasting approximately eight minutes. The interviewee’s performance in MMIs is usually not associated with cognitive, but rather with non-cognitive skills such as moral reasoning, study motivation and honesty, see, for example [13]. A properly designed MMI may thus also be used to obtain information on the willingness to work as country doctor.

One also could use waiting semesters as a signal for motivation. If an applicant is initially rejected, she has the option to wait for a semester to get a bonus on her high-school grade point average. Alternatively, she could start studying another subject. Some students spend their waiting semesters as health workers (e.g., nurses, medical specialists). We conjecture that those with high intrinsic motivation to become a medical doctor are more willing to wait for considerable time to be admitted and to be employed as health worker in the meantime^2^. Waiting semesters are already implemented in Germany, but only a small share of 20 percent of students is admitted because of this criterion.

Up to now, the success of entry requirements has mainly been discussed in terms of the percentage of students finishing their medical studies as well as their final grades. We suggest also including the specialization choice of students as a criterion when evaluating entry requirements.

Another measure to alter students’ specialization choice could be a change in the undergraduate medical curriculum. A recent observational study at Leipzig Medical School suggests that a highly practice-oriented family medicine curriculum may actually increase the share of graduates deciding to pursue postgraduate training as a general practitioner [14]. The presence of an independent institute and a chair for family medicine also seem to foster the motivation of medical students to consider a career as a general practitioner [15]. This is supported by [16] who surveyed final year medical students in their clinical elective in family medicine at seven German medical schools. When students were satisfied with the quality of this training they indicated a higher motivation to pursue a career in family medicine. 

Finally, postgraduate training itself and the professional profile of family medicine in Germany play a crucial role for the decision to become a general practitioner [17]: 89 percent of a nationally surveyed sample of German general practitioners consider to work in private practice and 77 percent can imagine to do so in a rural setting. Key factors for their willingness to settle as a general practitioner in a rural area were a family-friendly environment, the rural location itself, and the opportunity for collaboration with colleagues. 

## Limitations

One limitation of our study is that we only have students’ overall grade point average in our data. This score determines students’ chances of getting admitted to medical school. It consists of academic achievements in different disciplines such as math, languages, and science subjects. It would be important to know which disciplines drive our results, i.e., in which subjects those who are willing to practice in the countryside perform worse (or better) than the other students. Moreover, it would be important to know how important these subjects are for a physician’s daily business. If those disciplines in which future country doctors perform worse than the rest are not essential for their job performance, changing entry requirements to increase the supply of country doctors may not be costly in terms of physician quality.

Another limitation of our data is that we measure students’ professional preferences during their studies, not their subsequent actual professional choices. To evaluate the link between entry requirements for medical school and occupational choices, longitudinal survey data that allow researchers to follow individuals’ choices throughout their studies and the early stages of their professional careers would be very useful. In particular, this is important since medical students may change their career plans as they advance in their studies (see, for example [18]).

To study the role of origin for location choice it would have been important to ask students about whether they grew up in a city or in the countryside. Future studies may want to include such a question in their surveys

The response rates in our study are 25 percent (in 2012) and 15 percent (in 2014), respectively. Hence, our results are not representative for the total student population. A higher response rate would be desirable, but we note that the response rates we achieved are not atypical for social science survey as responses rates have declined considerably in recent years, see [19]. Moreover, it would be useful to have data from more than two universities. Finally, we only considered individuals studying to become a medical doctor. However, continuing education may also play a role for practicing physicians’ location choice. In recent years, this topic received increasing attention (see, for example [20]) since medical doctors have to keep track and take advantage of new technological developments in medicine.

## Policy perspectives

Policies to increase the supply of country doctors could exploit the fact that those willing to work in rural areas come from a different pool of applicants. Policy instruments such as multidimensional entry requirements for medical schools and the differentiation of the study program into different tracks may alleviate the shortage of rural doctors. In particular, grade requirements for admission could be lowered for candidates who have already worked in a health context or have a family background of rural medicine. A special curricular track for general practitioners could also prepare future physicians better for the requirements of the daily work as a general practitioner. In the economics literature, individuals with higher risk-aversion scores have been shown to be less willing to take risks regarding the variability of their incomes and to become self-employed. Since the prospective rural practitioners in our study are more risk averse, they should be supported during residency training and when starting their own practice as a general practitioner, for instance with targeted loan subsidies, help for networking with colleagues, and infrastructure support.

## Notes

^1^ A logit model would assume that the error terms follow an extreme value type II distribution. In general, the estimated effects of the logit and the probit model are very similar. This is also the case in our data. 

^2^ However, [21] find that students admitted after a positive number of waiting semesters performed significantly worse in their medical studies than those admitted immediately.

## Acknowledgements

We acknowledge the support by the German Science Foundation through SFB/TR 15. We thank Suzan Elshout and the team of programmers at CentERdata Tilburg. 

## Competing interests

The authors declare that they have no competing interests.

## Figures and Tables

**Table 1:**
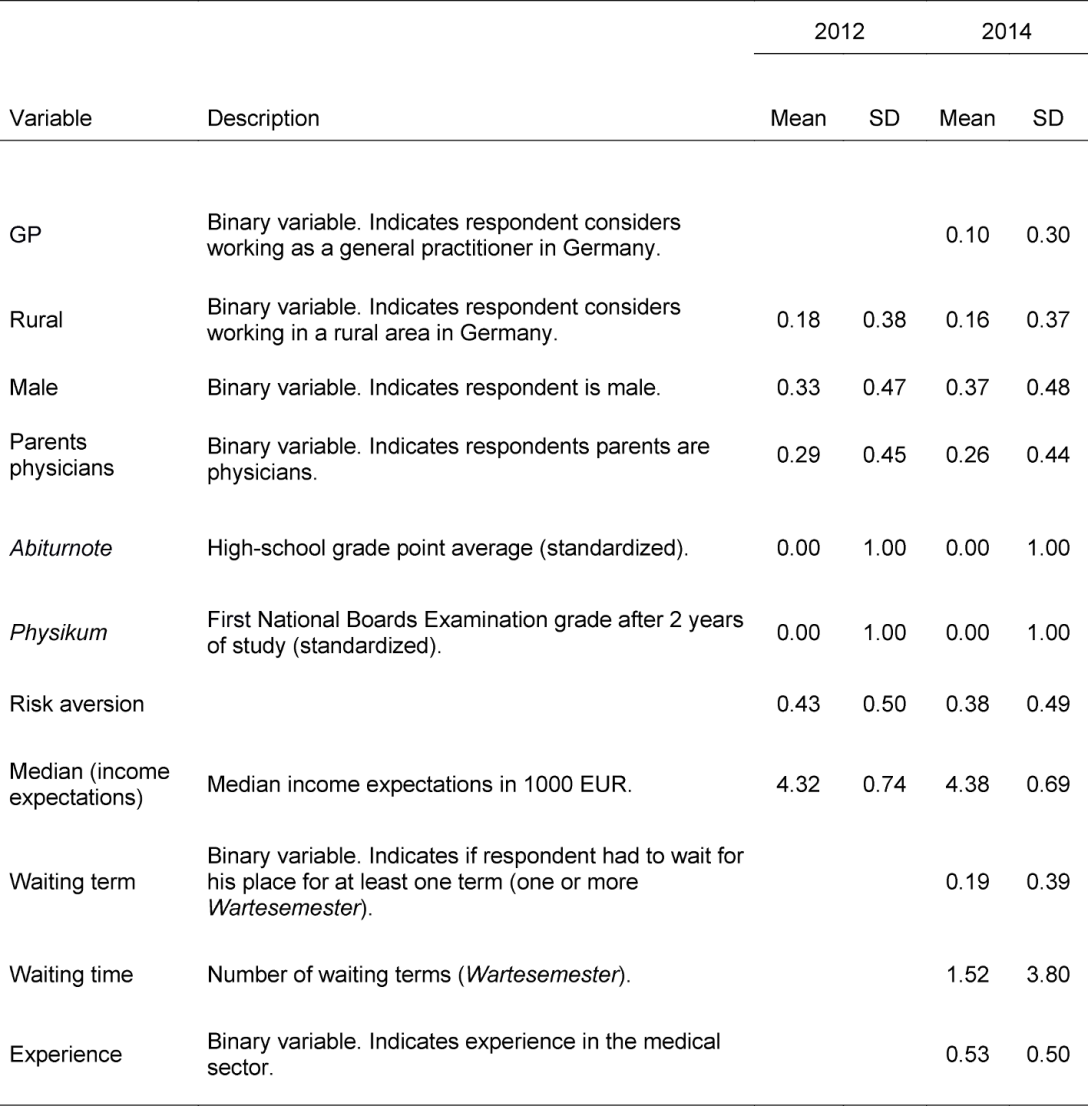
Variable definitions and descriptive statistics

**Table 2:**
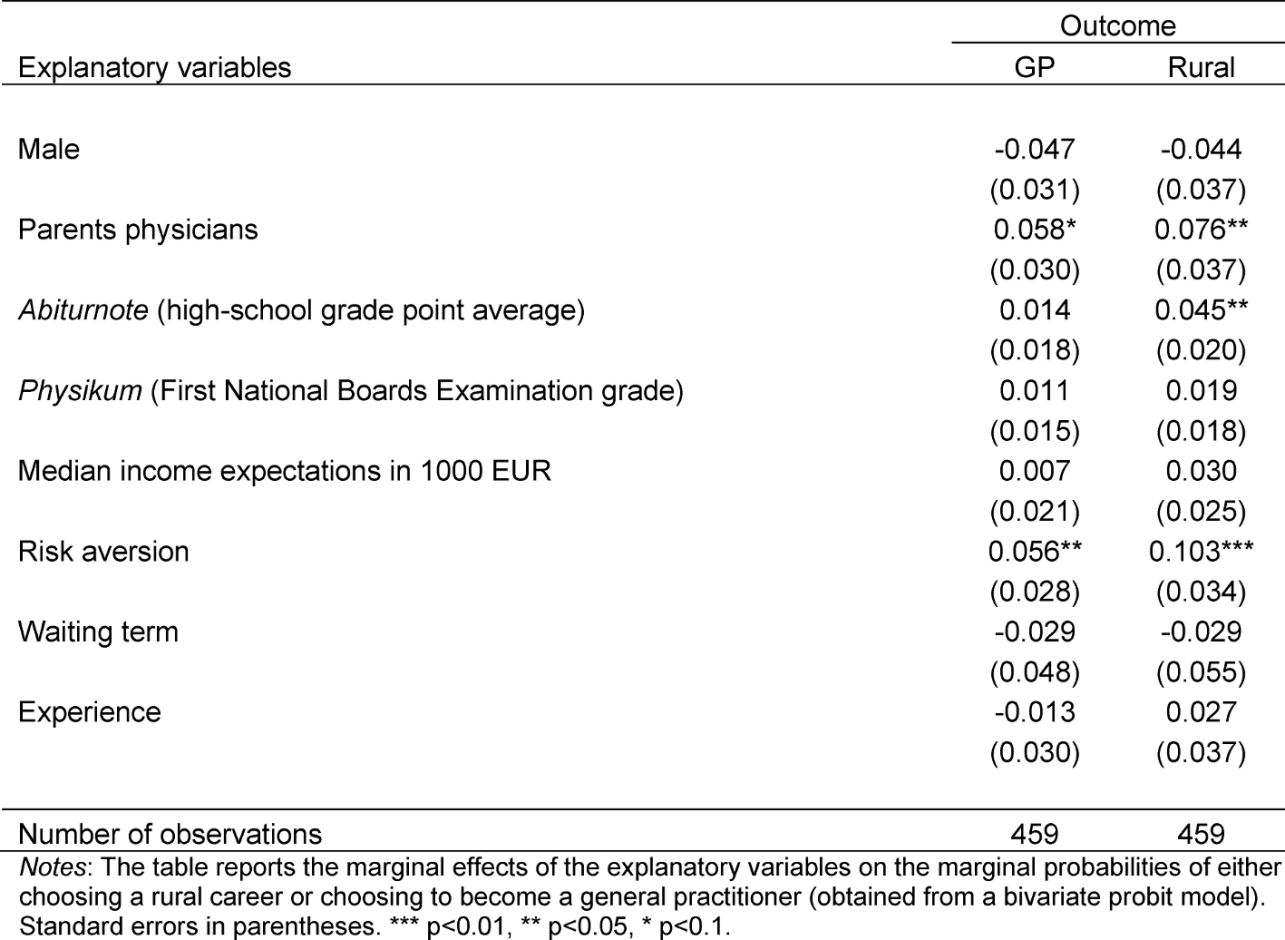
Multivariate regression results (marginal effects on marginal probabilities from bivariate probit)

**Table 3:**
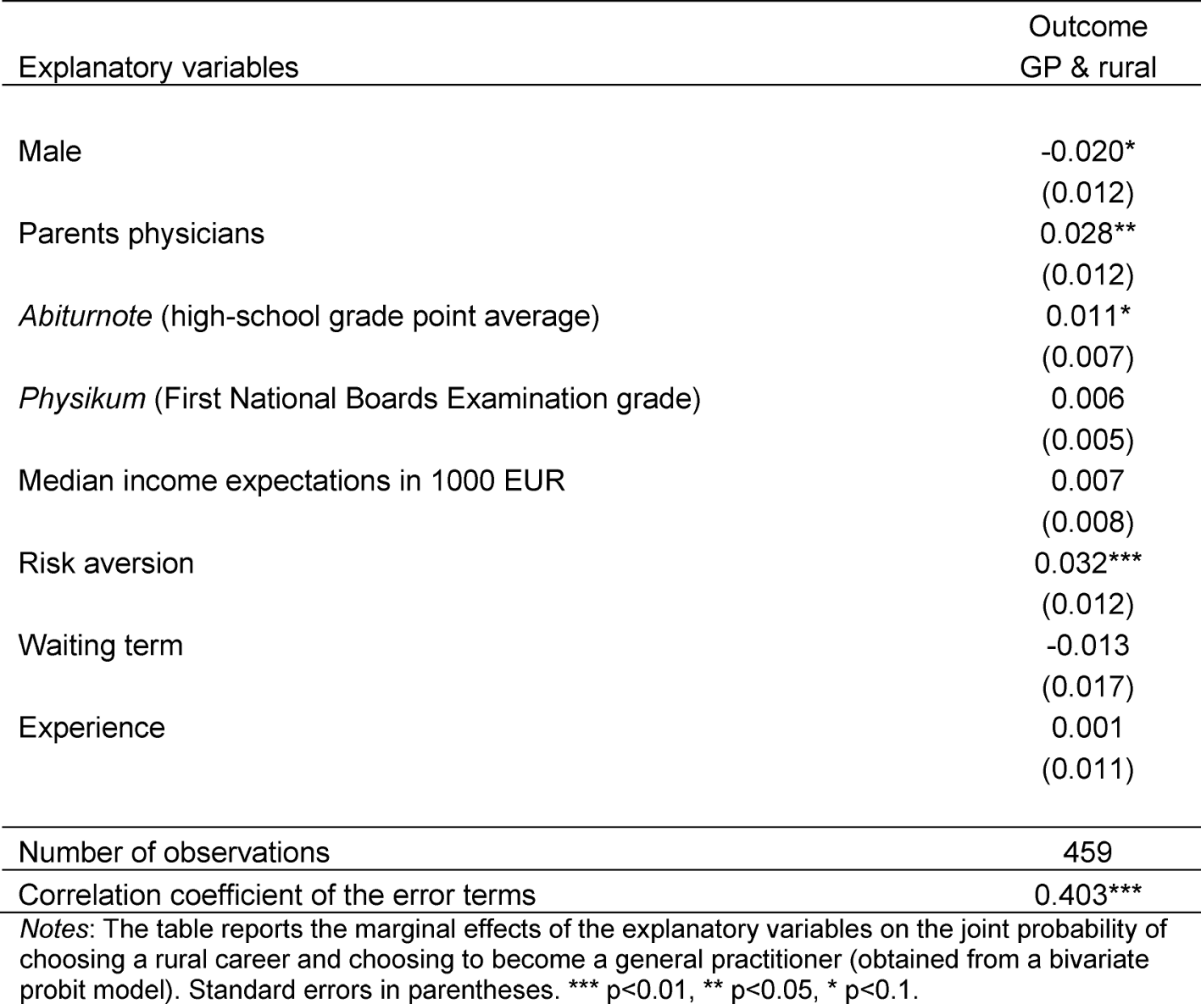
Multivariate regression results (marginal effects on joint probability from bivariate probit)
